# Increased Consumption of Unsaturated Fatty Acids Improves Body Composition in a Hypercholesterolemic Chinese Population

**DOI:** 10.3389/fnut.2022.869351

**Published:** 2022-04-25

**Authors:** Sumanto Haldar, Shalini Ponnalagu, Farhana Osman, Shia Lyn Tay, Long Hui Wong, Yuan Rong Jiang, Melvin Khee Shing Leow, Christiani Jeyakumar Henry

**Affiliations:** ^1^Clinical Nutrition Research Centre (CNRC), Singapore Institute of Food and Biotechnology Innovations (SIFBI), Agency for Science Technology and Research (A^*^STAR), Singapore, Singapore; ^2^WIL@NUS Corporate Laboratory, National University of Singapore, Centre for Translational Medicine, Singapore, Singapore; ^3^Wilmar (Shanghai) Biotechnology Research and Development Center Co., Ltd., Shanghai, China; ^4^Singapore Institute for Clinical Sciences (SICS), Agency for Science, Technology and Research (A^*^STAR), Brenner Centre for Molecular Medicine, Singapore, Singapore; ^5^Division of Medicine, Department of Endocrinology, Tan Tock Seng Hospital, Singapore, Singapore; ^6^Lee Kong Chian School of Medicine, Nanyang Technological University, Singapore, Singapore; ^7^Department of Biochemistry, National University of Singapore, 8 Medical Drive, Singapore, Singapore

**Keywords:** blended oils, olive oil, unsaturated fats, body fat, lean mass, DXA

## Abstract

While an increase in fat intake and the resulting excess calorie intake are implicated in weight gain, different fat types exert variable effects on body composition, with unsaturated fats showing favorable effects on body composition in Western population. Whether and to what extent these associations apply to Asian population have not been established. We investigated the effects of two separate Asian-based oil blends, rich in unsaturated fats, made from refined rice bran, sesame, and flaxseed oils, in comparison with refined olive oil, on body composition using dual-energy X-ray absorptiometry (DXA), from an 8-week, parallel design, randomized trial in 66 men (58.7 ± 5.71 years old, 23.0 ± 2.38 kg/m^2^) and 69 postmenopausal women (59.1 ± 5.34 years old, 21.7 ± 2.52 kg/m^2^), with borderline hypercholesterolemia. Despite increases in mean daily intakes of total energy (approximately +400 kcal/day, female, and approximately +240 kcal/day, male), as well as increases in percentage of calories from fats and proteins and decreases in percentage of calories from carbohydrates during the dietary intervention period, there were no significant changes in total body fat mass in both genders and also in all treatment groups. While total body weight increased slightly (0.36 ± 0.12 kg, *p* = 0.005) in women during intervention, this was mainly due to gain in lean mass (0.38 ± 0.081 kg, *p* < 0.0001). Correspondingly, there were reductions in total body fat (%), android fat (%), and gynoid fat (%) in women. No significant differences between the 3 intervention oil types were found in any of the measured parameters in either gender. Increasing relative intakes of unsaturated fats may prevent fat mass gain and circumvent muscle mass loss associated with menopause in older Asian women. Long-term studies are needed to confirm findings. This study had been registered on clinicaltrials.gov (Identifier No.: NCT03964857, https://www.clinicaltrials.gov/ct2/show/NCT03964857).

## Introduction

Dietary fats are generally associated with weight gain and obesity due to higher calorie density than carbohydrates and proteins (1, 2). However, obesity, particularly in Asians, is not necessarily caused by total dietary fat alone (3, 4), and the quality of fat also plays an important role (5). Different types of fatty acids have different metabolic outcomes such as fat oxidation and rate of deposition leading to different rates of fat mass gain (2). Fatty acid structure, chain length, degree of unsaturation, position, and configuration of double bonds all have been shown to affect the metabolic outcomes of different fatty acids (6–8). For example, a diet rich in MUFAs leads to reduced fat deposition as compared with SFAs (9), likely to be due to the preferential oxidation and metabolism of dietary MUFAs, thereby influencing body composition and reducing the risk of obesity (10). While several reviews in this area have indicated that unsaturated fats induce greater energy expenditure, fat oxidation, and diet-induced thermogenesis compared with saturated fatty acids (11, 12), the effect of the degree of unsaturation, in the form of MUFA or PUFA, has not been fully elucidated (11). Some clinical trials support that fish oils and in particular n-3 fatty acids can result in weight loss (13, 14) although other studies found no such effects (15, 16). These results are further supported by studies, which showed increases in muscle mass and function in healthy older adults with an n-3 PUFA-enriched diet (17); in addition, a systematic review of 18 studies shows that n-3 PUFA supplementation preserves strength and improves recovery in young, healthy adults (18). On the contrary, a separate 12 weeks study of PUFA supplementation in an elderly population with decreased muscle mass did not affect muscle mass, handgrip strength, or the timed “up-and-go” test (19). A possible reason for these equivocal findings with regard to PUFAs can be partly explained by the n-6:n-3 PUFA ratio in the diet rather than the absolute amounts of these FAs (20). While recent studies support the notion that consumption of n-6:n-3 PUFA ratio may be associated with longitudinal weight gain (21, 22), the majority of the above evidence came from Western population.

Given that Asians have different body composition patterning compared with Caucasians, including greater percentage of body fat, greater predisposition to abdominal adiposity, and lower fat-free mass, as well as worse consequences of the increased body fat toward cardiometabolic health outcomes in Asians (23), it is rather important to establish whether and which types of unsaturated fats have comparable desirable changes in body composition in Asians as that reported in Western population. We have recently shown improvements in blood lipid profile with an 8-week consumption of two types of blended oils (made from different proportions refined rice bran, flaxseed, and sesame seed oils), with effects comparable with refined olive oil (24). Since the three intervention diets had distinct fatty acid compositions, including PUFA:MUFA and n-6:n-3 PUFA ratios, in this report, we presented secondary analysis on detailed body composition data, using DXA measured at the beginning (week 0) and end (week 8) of the dietary intervention. Therefore, the objective of this secondary analysis was to investigate how the consumption of the two refined blended oils and refined olive oil in the diet for 8 weeks modulated several body composition parameters measured using DXA, separately for each gender. As exploratory analyses, we also investigated the correlation between changes in these body composition measures with changes in various blood biomarkers of cardiometabolic disease risk.

## Methods

The details of the study population and volunteer recruitment had been reported previously (24), but in brief, this study was undertaken in, Chinese men and menopausal women aged between 50 and 70 years, with a BMI ≤ 27.5 kg/m^2^ and with borderline hypercholesterolemia, defined as having serum LDL-cholesterol (LDL-C) between 3.06 and 4.51 mmol/L, based on ±10% of the NCEP ATPIII classification (25). This study had been registered on clinicaltrials.gov (Identifier No.: NCT03964857) and approved by a National Healthcare Group Domain Specific Review Board (NHG DSRB) ethics committee, Singapore (Reference: C/2018/00861).

This study was an 8-week, parallel design, randomized dietary intervention trial undertaken to test the effects of two different blended oils (blended oil 1 [BO1] and blended oil 2 [BO2]) and refined olive oil (ROO) as control. The BO1 and BO2 were prepared using refined rice bran oil (RBO), refined flaxseed oil, and refined sesame oil in predetermined ratios. The fatty acids and phytonutrient compositions of the three intervention oils have been reported previously (24) and summarized in Supplementary Table 1. Over the 8-week study period, volunteers were asked to consume 30 g/day of intervention oils through the provision of 2 meal accompaniments per day consisting of ready-made chicken dishes to be included as part of their daily diet. Each meal accompaniment contained 15 g of oil and approximately 18 g of protein per serving and came as 4 separate chicken dishes to ensure variety (i.e., braised chicken, chicken casserole, chicken curry, and chicken carrot stew) and increase compliance of volunteers to the intervention. Apart from the differences in oil contents, all other ingredients in the meal accompaniments and their preparation methods were identical between the three intervention groups. Volunteers were also asked to maintain their usual energy intake, habitual diet, and physical activity throughout the 8-week intervention period. Dietary intake was assessed every 2 weeks throughout the intervention using separate 3-day food diaries at weeks 0, 2, 4, 6, and 8, and the mean daily intakes of nutrients and energy were calculated using the FoodWorks software (version 10, Australia). Physical activity was also measured at the same times as the dietary assessments using the international physical activity questionnaire (IPAQ, long-form) as described in detail previously (24).

Finally, detailed body composition was measured using DXA (QDR Discovery Wi, fan-beam densitometer, Hologic, Waltham, MA, USA) on weeks 0 and 8. The software (version 8.21) provided by the manufacturer was used to generate the data. The “android region” was defined as the area between the ribs and the pelvis, whereas the “trunk” covered the entire region from the neck to the hips. The “gynoid region” included the hips and upper thighs, overlapping both the leg and trunk regions. Visceral fat was estimated using the machine's own software spanning L1–L5 regions. All metal items were required to be removed before measurement to prevent interference with the scanning process. While the whole body total bone mineral density (BMD) is presented to assess changes in BMD due to treatment and over time, the neck of femur bone mineral density (BMD) was measured for the categorization of individual bone health status. As such, *T* scores less than or equal to −2.5 are defined to be osteoporosis, *T* scores between −2.5 and −1 are defined to be osteopenia, and *T* scores greater than or equal to −1 are defined to be normal. The detailed methods for the measurements of blood markers were described previously (24). In brief, serum glucose and blood lipid panel were measured using an AU 5,800 clinical chemistry analyzer (Beckman Coulter, Inc., USA); apoA1 and apoB were measured using the immunoturbidimetric assay (Beckman Coulter 480); and insulin was measured using UniCel DxI 800 Access Immunoassay System (Beckman Coulter, Inc., USA).

### Statistical Analysis

Power calculation was originally performed on the primary outcome measures (i.e., blood lipid profile), as previously reported (24). All analyses reported here were performed separately for each gender, due to the inherent differences in lipid metabolism and body composition characteristics between men and women. Volunteers were randomly assigned to intervention groups using GraphPad prism, separately for men and women. Baseline comparisons between the three treatment groups were done using one-way ANOVA. The main outcomes of this exploration were the detailed body composition measurements obtained from DXA. Analysis of DXA body composition measurements was done using an intention-to-treat approach without imputing missing values. The linear mixed-effects model method in Statistical Package for the Social Sciences (SPSS) was used with treatment, time, and interaction between treatment and time as fixed effects for each gender separately. Compound symmetry (CS) covariance structure was used to model the within-participant correlation. The significant interaction between treatment and time was followed with a comparison within each treatment. The significant main effect of treatment was followed up with multiple pair-wise comparisons with Bonferroni correction. The same analysis was repeated for the nutrient data but with only those who have completed the entire 8 weeks of the dietary intervention (per-protocol, *N* = 128). The average intake during the 8-week intervention measured every 2 weeks was used to calculate the mean daily energy and nutrient intake during the dietary intervention period (to give a better indicator of average nutrient exposure over this period). Linear mixed-effects model method with CS covariance structure was also used to test for interaction of gender, treatment, and time on the overall combined data. The nutrient intake at week 0 was considered as “baseline” (i.e., pre-intervention habitual intake). Data were checked for normality visually using histogram and Quantile-Quantile (QQ) plot. Log transformation was done where necessary although untransformed raw values are presented in tables and figures for ease of interpretation.

Associations between the changes in body composition following dietary intervention (weeks 8–0) and the corresponding changes in lipid profile and other metabolic variables were evaluated using Spearman correlations (separately for each gender). Thus, these associations were undertaken in per-protocol population. One volunteer's data (randomized to BO2 treatment) were excluded from the lipid profile analyses throughout a previous study for being an outlier with large TG values (24). For consistency, the same volunteer was removed from the current analysis. The Sign test was used to test for differences in *T* scores categorization at baseline and end of trial within each intervention group. All statistical analyses were done using SPSS (IBM Corporation Released 2016. IBM SPSS Statistics for Windows, version 24.0. Armonk, NY: IBM Corporation), with α = 0.05.

## Results

All data are presented as mean ± standard error of the mean (SEM) unless stated otherwise. In total, 135 volunteers attended baseline measurements and received treatment allocation, with *n* = 44 in BO1, *n* = 44 in BO2, and *n* = 47 in ROO groups. Of those receiving an intervention, 1, 4, and 2 volunteers withdrew from BO1, BO2, and ROO groups, respectively. The reported mean compliance to the meal accompaniments, using the Intervention Food Records, were 98.9, 99.4, and 99.3% in ROO, BO1, and BO2 treatments, respectively, as measured in all volunteers who started the dietary intervention.

### Baseline Measurements

Body composition parameters measured immediately before the start of intervention at week 0 (baseline) using DXA are presented in Table 1, separately for each gender. There were no statistically significant differences in the baseline values between the treatment groups, following Bonferroni corrections.

**Table 1:** Baseline body composition measurements obtained using DXA of the participants randomized into the three intervention groups, namely, BO1, BO2, and ROO, presented as mean ± standard error of the mean.

**Variable**	**BO1 (*****n*** **=** **44)**		**BO2 (*****n*** **=** **44)**		**ROO (*****n*** **=** **47)**	
	**Males (*n* = 23)**	**Females (*n* = 21)**	**Males (*n* = 21)**	**Females (*n* = 23)**	**Males (*n* = 22)**	**Females (*n* = 25)**
Total mass (kg)	63.6 ±1.80	53.3 ± 1.72	65.1 ± 1.33	50.9 ± 1.01	65.0 ±2.18	54.1 ± 1.73
Total fat mass (kg)	17.9 ± 0.91	20.4 ± 1.24	18.1 ± 0.71	19.3 ± 0.77	16.5 ± 1.10	20.7 ± 0.87
Visceral fat mass (g)	493 ± 36.4	444 ± 53.1	534 ± 37.1	382 ± 33.0	469 ± 42.0	429 ± 35.9
Total fat (%)	28.1 ± 1.12	37.7 ± 1.40	27.6 ± 0.79	37.6 ± 0.94	24.8 ± 1.06	38.2 ± 0.98
Android fat (%)	32.8 ± 1.49	38.7 ± 2.08	34.0 ± 1.22	37.6 ± 1.47	29.8 ± 1.57	39.9 ± 1.31
Gynoid fat (%)	29.5 ± 1.25	42.2 ± 1.22	29.1 ± 0.74	42.6 ± 0.69	25.8 ± 1.11	42.9 ± 1.07
Android fat% to gynoid fat% ratio	1.12 ± 0.040	0.91 ±0.035	1.17 ± 0.035	0.88 ± 0.031	1.15 ± 0.035	0.94 ± 0.032
Trunk fat (%)	30.2 ± 1.18	37.2 ± 1.82	30.1 ± 1.06	36.6 ±1.26	26.8 ± 1.31	38.1 ± 1.18
Trunk fat% / leg fat% ratio	1.18 ± 0.043	0.91 ± 0.031	1.19 ± 0.036	0.90 ± 0.029	1.17 ± 0.032	0.96 ± 0.031
Total lean mass (kg)	43.4 ± 1.31	31.2 ± 0.86	44.8 ± 0.93	30.0 ± 0.47	46.2 ± 1.25	31.5 ± 1.14
Whole body total bone mineral	1.10 ± 0.022	1.01 ± 0.025	1.09 ± 0.024	0.97 ± 0.016	1.12 ± 0.028	1.03 ± 0.023
density (BMD)^3^

*One-way ANOVA was used to test for differences in the mean between the treatment groups separately for each gender, and there were no significant differences between groups after Bonferroni corrections*.

### Food Diary Analysis

The mean intakes of energy and nutrients during the 8-week dietary intervention period (weeks 1–8) as compared with the volunteers' habitual intake at baseline (week 0) are presented in Tables 2, 3 for women and men, respectively. Compared with baseline, in both women and men, there were significant increases in total energy intake overall and in particular percentage of energy from both fats and proteins during the intervention period. On the contrary, there were significant decreases in the intake of percentage of energy from carbohydrates in both groups during the intervention group, compared with baseline. Time by treatment interactions for some of these variables was significant, as shown in the breakdown of various treatment groups (Tables 2, 3). As for other nutrients, potassium intake was reduced in both genders during the intervention period and fiber intake was reduced only in men.

**Table 2:** The nutrient data as mean ± standard error of the mean for the baseline and during intervention (week 8 post; which is the average of the post-baseline values) for each of the intervention groups in women, including only those who have completed the entire intervention (*N* = 66; ROO = 24, BO1 = 20, BO2 = 22).

**Variables**	**Pooled time**	**Week 0**	**Week 8 post**	***P*-value (time)**	***P*-value (treatment)**	***P*-value (interaction)**
Energy intake (kcal/d)				**<0.0001**	0.766	**0.006**
Pooled treatment		1,720 ± 58.8	2,120 ± 60.2			
*ROO*	1,980 ± 80.6	1,650 ± 96.2	2,310 ± 88.3*			
*BO1*	1,870 ± 69.9	1,770 ± 97.2	1,960 ± 98.1			
*BO2*	1,910 ± 83.6	1,750 ± 113	2,070 ± 116*			
Carbohydrate (% kcal)				**<0.0001**	0.17	**0.038**
Pooled treatment		48.0 ±0.85	40.2 ±0.75			
*ROO*	42.8 ± 1.18	48.1 ± 1.39	37.5 ± 1.14^*b^			
*BO1*	43.9 ± 1.20	47.1 ± 1.54	40.6 ± 1.57^*a, b^			
*BO2*	45.8 ±1.0	48.7 ±1.53	42.9 ± 0.97^*a^			
Protein (% kcal)		18.6 ± 0.50	20.0 ± 0.24	**0.005**	0.291	0.843
Fat (% kcal)				**<0.0001**	0.117	**0.022**
Pooled treatment		33.4 ±0.74	39.7 ±0.65			
*ROO*	38.2 ± 0.99	33.7 ± 1.23	42.6 ± 0.88^*a^			
*BO1*	36.1 ± 1.07	33.4 ± 1.39	38.9 ± 1.40^*a, b^			
*BO2*	35.3 ± 0.83	33.1 ± 1.32	37.4 ± 0.80^*b^			
Dietary fiber (g/d)		23.1 ± 1.37	23.0 ± 1.04	0.686	0.527	0.566
Sodium (g/d)		2.98 ± 0.47	2.34 ± 0.11	0.336	0.189	0.577
Potassium (g/d)		1.55 ± 0.068	1.36 ± 0.14	**0.001**	0.438	0.800

*Untransformed data are presented for ease of interpretation. p-value = 0.05;*

^*^indicates columns that were significantly different (p-value < 0.05) from each other in each row. Different alphabet superscripts (“a, b”) within a column indicate treatment groups that were significantly different from each other. Bold values indicate p < 0.05.

**Table 3:** Nutrient data as mean ± standard error of the mean for the baseline and during intervention (week 8 post; which is the average of the post-baseline values) for each of the intervention groups in men, including only those who have completed the entire intervention (*N* = 62; ROO = 21, BO1 = 23, BO2 = 18).

**Variables**	**Pooled time**	**Week 0**	**Week 8 post**	***P*-value (time)**	***P*-value (treatment)**	***P*-value (interaction)**
Energy intake (kcal/d)				**<0.001**	0.313	0.521
Pooled treatment		2090 ± 105	2330 ± 70.3			
*ROO*	2340 ± 146	2250 ± 268	2500 ± 117			
*BO1*	2180 ± 95.7	2030 ± 128	2340 ± 138			
*BO2*	2040 ± 59.9	1970 ± 87.9	2120 ± 79.9			
Carbohydrate (% kcal)				**<0.0001**	0.813	0.384
Pooled treatment		48.3 ± 1.10	41.9 ± 0.80			
*ROO*	44.8 ± 1.41	48.9 ± 2.21	40.7 ± 1.28			
*BO1*	44.8 ± 1.0	47.4 ± 1.50	42.2 ± 1.10			
*BO2*	46.0 ± 1.48	48.9 ± 2.13	43.0 ± 1.85			
Protein (% kcal)		18.1 ± 0.42	20.1 ± 0.34	**<0.0001**	0.373	0.100
Fat (% kcal)				**<0.0001**	0.505	0.508
Pooled treatment		33.6 ± 1.01	38.0 ± 0.64			
*ROO*	36.8 ± 1.17	34.2 ± 1.95	39.4 ± 1.06			
*BO1*	35.8 ± 0.88	34.2 ± 1.43	37.4 ±0.95			
*BO2*	34.6 ± 1.23	32.1 ± 1.93	37.0 ± 1.35			
Dietary fiber (g/d)		27.7 ± 2.34	23.8 ± 1.53	**0.019**	0.686	0.317
Sodium (g/d)		3.24 ± 0.25	3.17 ± 0.22	0.886	0.274	0.751
Potassium (g/d)		1.75± 0.23	1.32 ± 0.0932	**0.004**	0.444	0.806

*Untransformed data are presented for ease of interpretation. Bold values indicate p < 0.05*.

### DXA Body Composition Measurements

The main effects of time, treatment, and their interaction are presented in Tables 4, 5 for women and men, respectively. In women, pooled over the treatment groups, there were statistically significant increases in body weight (0.36 kg; 95% CI: 0.12 kg, 0.60 kg; *p*-value = 0.005) and total lean mass (mean gain = 0.38 kg; 95% CI: 0.22 kg, 0.54 kg; *p*-value < 0.0001) over the course of the dietary intervention (i.e., time effect). Correspondingly, pooled over the treatment groups, there were statistically significant decreases in total body fat percentage (total fat [%]) (−0.29%; 95% CI: −0.49, −0.082%; *p*-value= 0.007), android fat percentage (−0.65%; 95% CI: −1.12, −0.18%; *p*-value= 0.008), and gynoid fat (%) (−0.25%; 95% CI: −0.49, −0.010%; *p*-value= 0.042). The effect of treatment or treatment by time interaction was not significant in women. In men, on the contrary, none of the time, treatment, or time by treatment interaction comparisons was statistically significant. We have also analyzed the overall dataset with two genders pooled, which is presented in Supplementary Table 2.

**Table 4:** Baseline and end of intervention (week 8) body composition measurements obtained using DXA in women.

**Variables**	**Week 0**	**Week 8**	***P*-value (time)**	***P*-value (treatment)**	***P*-value (interaction)**
Total body mass (kg)	52.8 ± 0.89	53.1 ± 0.91	**0.005**	0.355	0.241
Total fat mass (kg)	20.1 ± 0.55	20.1 ± 0.57	0.799	0.585	0.249
Visceral fat mass (g)	418 ± 23.3	409 ± 23.5	0.174	0.624	0.409
Total fat (%)	37.9 ± 0.63	37.5 ± 0.64	**0.007**	0.906	0.544
Android fat (%)	38.8 ± 0.92	38.1 ±0.95	**0.008**	0.607	0.662
Gynoid fat (%)	42.6 ± 0.58	42.2 ± 0.60	**0.042**	0.898	0.796
Android fat % to gynoid fat % ratio	0.91 ± 0.019	0.90 ± 0.019	0.060	0.470	0.564
Trunk fat (%)	37.3 ± 0.81	37.1 ± 0.79	0.558	0.668	0.441
Total lean mass (kg)	30.9 ± 0.51	31.3 ± 0.52	**<0.0001**	0.476	0.474
Whole body total BMD (g/cm^2^)	1.00 ± 0.013	1.00 ± 0.013	0.13	0.15	0.49

*Data are presented as mean ± standard error of the mean. Data are pooled over treatment groups at each time point. Bold values indicate p < 0.05*.

**Table 5:** Baseline and end of intervention (week 8) body composition measurements obtained using DXA in men.

**Variables**	**Week 0**	**Week 8**	***P*-value (time)**	***P*-value (treatment)**	***P*-value (interaction)**
Total body mass (kg)	64.6 ± 1.04	64.8 ± 1.05	0.098	0.811	0.490
Total fat mass (kg)	17.5 ± 0.53	17.4 ± 0.54	0.867	0.430	0.671
Visceral fat mass (g)	498 ± 22.2	488 ± 20.4	0.749	0.556	0.761
Total fat (%)	26.9 ± 0.60	26.6 ± 0.61	0.508	0.071	0.362
Android fat (%)	32.2 ± 0.85	31.9 ± 0.87	0.692	0.137	0.360
Gynoid fat (%)	28.1 ± 0.64	27.9 ± 0.64	0.492	0.074	0.056
Android fat % to gynoid fat % ratio	1.15 ± 0.021	1.14 ± 0.023	0.989	0.551	0.365
Trunk fat (%)	29.0 ± 0.71	29.0 ± 0.72	0.284	0.093	0.259
Total lean mass (kg)	44.8 ± 0.69	45.1 ± 0.71	0.083	0.309	0.230
Whole body total BMD (g/cm^2^)	1.10 ± 0.014	1.11 ± 0.014	0.78	0.65	0.73

*Data are presented as mean ± standard error of the mean. Data are pooled over treatment groups at each time point*.

### Correlations Between Changes in Body Composition Measures Due to Intervention With Changes in Blood Markers of Cardiometabolic Health

Considering that there was rarely any time by treatment interactions or effects of treatment groups *per se* (Tables 4, 5), we pooled all subjects from all three treatment groups together to ascertain the associations between changes in body composition over time and the corresponding changes in metabolic variables in blood, separately for each gender group, as presented in Tables 6, 7 respectively.

**Table 6:** Spearman's correlations between changes in body composition measures due to intervention with changes in blood markers of cardiometabolic health in women (*N* = 66).

			**Total**			**LDLC**	**Total Cholesterol**	**LDLC**			**ApoB to**
	**Insulin**	**Glucose**	**Cholesterol**	**Triglyceride**	**HDLC**	**calculated**	**to HDL ratio**	**measured**	**ApoA1**	**ApoB**	**ApoA1 ratio**
Total fat mass	0.018	0.101	−0.206	0.230	−0.076	−0.274^*^	−0.101	−0.258^*^	−0.050	−0.256^*^	−0.164
Visceral fat mass	0.148	0.061	−0.062	0.400^**^	−0.204	−0.134	0.119	−0.060	−0.096	−0.080	0.006
Total fat (%)	−0.082	0.082	−0.126	0.090	−0.020	−0.171	−0.096	−0.174	−0.120	−0.180	−0.067
Android fat (%)	−0.016	0.015	0.005	0.256^*^	−0.109	−0.032	0.085	−0.031	−0.012	−0.036	−0.010
Gynoid fat (%)	−0.282^*^	−0.073	−0.061	−0.090	−0.015	−0.018	−0.046	−0.063	−0.142	−0.075	0.005
Android fat % to	0.144	0.080	0.042	0.331^**^	−0.099	−0.033	0.107	0.001	0.094	−0.008	−0.042
gynoid fat % ratio											
Trunk fat (%)	−0.110	0.057	−0.153	0.183	−0.023	−0.225	−0.122	−0.178	−0.033	−0.237	−0.161
Trunk fat % leg	0.005	0.146	−0.130	0.291^*^	−0.039	−0.202	−0.065	−0.119	0.026	−0.240	−0.176
fat % ratio											
Total lean mass	0.209	−0.068	−0.131	0.191	−0.039	−0.198	−0.072	−0.136	0.123	−0.140	−0.209

* *indicates p-values < 0.05,*

** *indicate p-values < 0.01*.

**Table 7:** Spearman's correlations between changes in body composition measures due to intervention with changes in blood markers of cardiometabolic health in men (*N* = 61, one subject was excluded from analysis as lipid variables were too extreme).

			**Total**			**LDLC**	**Total Cholesterol**	**LDLC**			**ApoB to**
	**Insulin**	**Glucose**	**Cholesterol**	**Triglyceride**	**HDLC**	**calculated**	**to HDL ratio**	**measured**	**ApoA1**	**ApoB**	**ApoA1 ratio**
Total fat mass	−0.12	0.073	−0.027	0.078	−0.093	−0.053	0.052	−0.002	−0.020	0.037	0.104
Visceral fat mass	0.043	0.122	−0.076	−0.060	−0.055	−0.119	−0.024	−0.114	0.005	−0.058	−0.031
Total fat (%)	−0.204	0.043	0.072	0.043	−0.024	0.055	0.091	0.097	−0.001	0.149	0.189
Android fat (%)	−0.035	0.028	0.073	0.123	−0.073	0.048	0.166	0.096	0.030	0.156	0.165
Gynoid fat (%)	−0.159	−0.016	0.025	0.041	−0.165	0.057	0.169	0.039	−0.177	0.104	0.228
Android fat % to	0.059	0.03	−0.010	0.052	0.097	−0.065	−0.067	0.023	0.136	0.015	−0.059
gynoid fat % ratio											
Trunk percent fat	−0.254^*^	0.03	0.001	0.102	−0.104	−0.024	0.077	0.026	−0.052	0.084	0.175
Trunk fat % leg	−0.027	0.075	−0.157	0.001	−0.045	−0.190	−0.184	−0.134	−0.043	−0.115	−0.057
fat % ratio											
Total lean mass	0.216	0.100	−0.209	−0.055	−0.012	−0.199	−0.194	−0.210	−0.002	−0.270^*^	−0.254^*^

* *indicates p-values < 0.05,*

^**^indicate p-values < 0.01.

Changes in visceral fat mass (*r*_s_ = 0.40, *p*-value < 0.05), android fat (%) (*r*_s_ = 0.26, *p*-value < 0.05), android fat % to gynoid fat % ratio (*r*_s_ = 0.33, *p*-value < 0.05), and % fat in trunk/% fat in legs ratio (%) *r*_s_ = 0.29, *p*-value < 0.05) were positively associated with changes in TG concentration in women. On the contrary, changes in total fat mass were negatively associated with LDL-C (*r*_s_ = −0.27, *p*-value < 0.05) and Apo B (*r*_s_ = −0.26, *p*-value < 0.05) concentrations in women. There was also a negative association between changes in gynoid fat % and insulin concentration (*r*_s_ = −0.28, *p*-value < 0.05) in women. Changes in total lean mass was negatively associated with serum Apo B concentration (*r*_s_ = −0.27, *p*-value < 0.05) in men, and change in percentage of trunk fat was negatively associated with fasting serum insulin concentration (*r*_s_ = −0.25, *p*-value < 0.05) in men.

## Discussion

We have previously reported significant improvements in blood lipid profile and blood pressure, despite a small but significant weight gain of approximately 0.42 kg ± 0.11 kg (*p* < 0.05), in both genders combined (24). In this secondary analysis, we reported detailed body composition analyses using DXA and found that following the 8-week intervention period, despite the reported average excess calorie intakes of 400 and 240 kcal/day in women and men, respectively, there was no significant increase in total fat mass or visceral fat mass in either gender. The excess calories in both genders came predominantly from the proteins and fats in the intervention foods, which the volunteers were adding on top of their habitual diet, rather than replacing them for other protein and fat sources in their diet, as previously reported. Our intervention foods that contained 30 g of intervention oils (either ROO, BO1, or BO2) and 36 g of protein per day also contributed to the greater percentage of calories from fats and proteins and lower percentage of calories from carbohydrates during the intervention period. Therefore, the fat types in our intervention foods, which were predominantly MUFAs and PUFAs (24), may have contributed to the lack of either fat mass or visceral fat mass gain in both genders. In fact, previous reports have shown that increased relative intake of linoleic acid was found to be inversely associated with visceral adipose tissue and trunk fat, whereas palmitic acid was less consistently associated with body fat storage (26, 27). Another study by Alves et al. also showed that regular intake of high-oleic peanuts was found to improve fat oxidation and body composition in overweight men following an energy-restricted diet (28) with body fat % being significantly reduced in those who consumed high-oleic peanuts while the total percentage lean mass also increased. A separate study in an insulin-resistant population consuming a diet rich in MUFAs also showed significantly increased fat oxidation rates and lower abdomen-to-leg adipose ratios than carbohydrate-rich diets, thus preventing visceral adiposity and corresponding improvements in adiponectin and insulin sensitivity as compared with a carbohydrate-rich diet (29).

Thus in Asia, where individuals have a greater proportion of body fat mass and visceral fat mass compared with the Caucasian population, with Asians being more prone to insulin resistance, prediabetes, and type 2 diabetes (23), choosing the right type of vegetable oils may form a key strategy to improve metabolic health. Due to the limitations in the food composition databases of Asian foods and the variety of oils used in Asian cuisine, we were unable to accurately quantitate the fatty acid intake profile of the entire diet either at baseline or during the intervention. However, we can assume that during the intervention period, the relative proportion of unsaturated fats in the diet increased compared with total saturated fats. As such, many studies have established that dietary displacement of saturated fats with various types of unsaturated fats is likely to improve body composition, including body fat proportion and distribution. For example, large prospective observational studies indicated that both MUFA and PUFA were not associated with weight gain (5, 30), whereas another cross-sectional study found similar results only for PUFA (31). Similarly, Piers et al. substituted a diet rich in SFA with MUFA for a period of 4 weeks in 8 obese men with a randomized cross-over study design to investigate the effects on body weight and composition (32). Using DXA, the authors found a small but significant decrease in body mass (−2.1 ± 0.4 kg; *p* = 0.0015) and fat mass (−2.6 ± 0.6 kg; *p* = 0.0034) in those who followed the MUFA rich diet, as compared to SFA, even though there was no difference in total energy intake or fat intake (32). Furthermore, there was also a decrease in the waist-to-hip ratio of those who consumed the MUFA-rich diet, and these improvements in body composition were also later observed in normal-weight individuals (32, 33). Another RCT also showed reductions in abdominal adiposity after 10-week supplementation with PUFA rich foods as compared with SFA-rich foods (34).

We also observed that during the intervention, any weight gain, particularly in women, was mainly associated with increases in lean/muscle mass. While the muscle mass gain was not statistically significant in men (mean increase of approximately 0.3 kg, *p* = 0.083), this was significant in women (mean increase of approximately 0.4 kg, *p* < 0.0001), with this also explaining significant reductions in total fat (%), android fat (%), and gynoid fat (%) in women during the course of the dietary intervention. This increase in muscle mass in the female groups *per se* was likely to be due to the combination of the increases in protein intake along with the relative and absolute increase in the intake of unsaturated fats. An increase in lean mass with unsaturated fatty acids has also been shown previously, including a dietary intervention study with linoleic acid (18:2n-6) from safflower oil, which showed increases in lean mass after 16 weeks (35). Another short-term 7-week study comparing PUFA rich sunflower oil intervention as compared with SFA-rich palm oil also showed significantly greater improvements in lean tissue in the PUFA rich diet (36). Similarly, a cross-sectional study in an older cohort also observed that plasma levels of PUFA were associated with larger muscle size (37). Given that the increase in muscle mass in our female volunteers occurred without any enhancement of physical activity [data not shown, (24)] and within a relatively shorter time frame, the increases in the intake of dietary unsaturated fats may be one of the strategies for postmenopausal women, who are at greater risk of muscle mass loss following menopause (38, 39). It is also interesting to observe that we did not find any differences in effects between intervention oil types (treatment effects) or any time by treatment effects in the majority of the parameters measured in both genders. This may be mainly because either the addition of unsaturated fats to diet and/or replacement of saturated fats with unsaturated fats had a greater effect than that between the different types of unsaturated fats in the 3 intervention oils used in our study. As expected, while there were no changes in bone mineral density (BMD) throughout the intervention, we noticed a significant proportion of the study population (>50%) suffering from either osteopenia or osteoporosis (Supplementary Table 2). Therefore, more studies need to be undertaken to establish how diet and lifestyle can improve bone health in Asian population.

Finally, correlations between intervention-related changes in body composition measures and various blood markers of cardiometabolic health enabled us to explore how these parameters may be inter-related. The positive association of visceral fat mass, android fat%, and android fat% to gynoid fat% ratio with fasting plasma TG in women have been long established. However, the lack of such significance in men was somewhat unexpected and highlights the possible phenotypic differences between genders in metabolic health parameters. The negative association between changes in total fat mass with LDL-C and Apo B concentration in women also requires further exploration but the findings highlight that fat distribution, rather than total body fat amount may be more relevant with regards to cardiovascular disease (CVD) risbetween changes in total fatk. This has been supported by several larger epidemiological studies, whereby elevated trunk fat that was associated with increased CVD risk, whereas the associations were opposite for arm/leg fat (40, 41). Given that postmenopausal women are more likely to have a greater proportion of fats as peripheral fats, as opposed to the viscera (42), this partly explains our finding. Interestingly, the negative association between gynoid fat (%) and fasting insulin in women also supports other findings, including one study in obese adolescents showing HOMA-IR value having a significant positive association with android to gynoid fat ratio (%) and another more recent cross-sectional study showing peripheral fat, including gynoid fat being protective against insulin resistance in both women and men (43). However, these same associations were non-significant in men. In fact, the significant negative association between changes in trunk fat % and the changes in fasting serum insulin in men was somewhat surprising given that previous studies have shown the markers of insulin resistance (HOMA-IR) is in fact positively associated with visceral/abdominal fat (44). The negative association between changes in lean mass and changes in Apo B indicates further beneficial effects of lean mass on cardiometabolic health.

In summary, despite a greater intake of total energy (including increases in percentage of calories from fats and proteins and reduction in percentage of energy from carbohydrates), it is likely that the relative and absolute increases in the intakes of both polyunsaturated and monounsaturated fats contributed toward no significant total or visceral fat mass gain and the relative increases in lean/muscle mass in women. This indicates that the quality of fats plays an important role in body composition and in particular body fat partitioning and distribution. Given that aging is generally associated with increases in fat mass and decrease in muscle mass, our study showed that consumption of appropriately formulated oil blends, originating in Asia, can improve body composition in older individuals. The lack of difference between the oil types, which were distinct in the types and ratios of various unsaturated fats, may indicate comparable, favorable effects of unsaturated fats, irrespective of the degrees of saturation, and/or chain lengths of fatty acids. Finally, the significant correlations between changes in body composition measures with changes in various metabolic measures further highlight that the types of body fat have different metabolic consequences. The strength of the study is that it is one of the few randomized dietary interventions that explored associations between increased intake of various types of unsaturated fatty acids and detailed body composition parameters using DXA in an older Asian population. There were also several limitations of this study, including the limited information on the fatty acid intake of the total diet and the relatively shorter duration (8 weeks) of the dietary intervention. Therefore, long-term studies in larger population are warranted to confirm the findings.

## Data Availability Statement

The original contributions presented in the study are included in the article/Supplementary Material, further inquiries can be directed to the corresponding author/s.

## Ethics Statement

The studies involving human participants were reviewed and approved by National Healthcare Group Domain Specific Review Board (NHG DSRB) Ethics Committee (Domain C), Singapore (Reference: C/2018/00861). The patients/participants provided their written informed consent to participate in this study.

## Author Contributions

CH, SH, ML, and YJ designed the research. SH, LW, ST, and FO enrolled volunteers and conducted the research. SP generated the random allocation sequence. SP, SH, and FO analyzed data or performed statistical analysis, wrote the manuscript and all authors read and approved the final manuscript.

## Funding

This project has been jointly funded by the National University of Singapore, Agency of Science Technology and Research (A*STAR), Singapore, and Wilmar International Limited.

## Conflict of Interest

LW and YJ are the current employees of Wilmar International Limited. The remaining authors declare that the research was conducted in the absence of any commercial or financial relationships that could be construed as a potential conflict of interest.

## Publisher's Note

All claims expressed in this article are solely those of the authors and do not necessarily represent those of their affiliated organizations, or those of the publisher, the editors and the reviewers. Any product that may be evaluated in this article, or claim that may be made by its manufacturer, is not guaranteed or endorsed by the publisher.
