# Supplementary material for: Increased Consumption of Unsaturated Fatty Acids Improves Body Composition in a Hypercholesterolemic Chinese Population
Source: Front Nutr. 2022 Apr 25;9:869351. doi: 10.3389/fnut.2022.869351 (PMC9082591; doi:10.3389/fnut.2022.869351)
Supplement: Supplementary file 1 [file Table_1.docx]

Supplementary Material

**Supplementary Table 1:** The composition and ratio of fatty acids in the 3 intervention oils

|  | **ROO** | **BO1** | **BO2** |
| --- | --- | --- | --- |
| **Fatty Acid Composition (% of total fatty acids)** | |  |  |
| Saturated Fat, SFA | 16.6 | 14.9 | 13.6 |
| Monounsaturated Fat, MUFA | 70.8 | 32.9 | 28.1 |
| Polyunsaturated Fat, PUFA n-6 | 11.5 | 30.8 | 23.9 |
| Polyunsaturated Fat, PUFA n-3 | 1.0 | 21.4 | 34.5 |
| MUFA : SAFA ratio | 4.26 | 2.21 | 2.07 |
| PUFA : SAFA ratio | 0.75 | 3.50 | 4.29 |
| PUFA n-6 : PUFA n-3 ratio | 11.5 | 1.4 | 0.7 |
| Phytosterol (mg/kg) | 1780 | 7600 | 5700 |
| Squalene (mg/kg) | 1480 | N.A. | N.A. |
| Tocopherol (mg/kg) | 186 | 325 | 315 |
| Olive Polyphenol (mg/kg) | 0 | N.A. | N.A. |
| Oryzanol (mg/kg) | N.A. | 8000 | 4800 |
| Sesamin (mg/kg) | N.A. | 720 | 300 |
| Sesamolin (mg/kg) | N.A. | 300 | 125 |
|  |  |  |  |
| ROO: Refined Olive Oil, BO1: Blended Oil 1 and BO2: Blended Oil 2 | | |  |

**Supplementary Table 2.** Presents baseline and end of intervention (week 8) body composition measurements obtained using DXA in the overall dataset. Data are presented as mean ± standard error of the mean. Data is pooled over treatment groups at each time point.

| Variables | Week 0 | Week 8 | P value (time) | P value (treatment) | P value (interaction)^1^ | P value (gender interaction)^2^ |
| --- | --- | --- | --- | --- | --- | --- |
| Total body mass (kg) | 58.5 ± 0.85 | 58.8 ± 0.86 | **0.001** | 0.81 | 0.26 | 0.47 |
| Total fat mass (kg) | 18.8 ± 0.40 | 18.8 ± 0.41 | 0.99 | 0.95 | 0.22 | 0.89 |
| Visceral fat mass (g) | 457 ± 16.4 | 447 ± 16.0 | 0.27 | 0.90 | 0.79 | 0.42 |
| Total fat (%) | 32.5 ± 0.64 | 32.2 ± 0.65 | **0.02** | 0.85 | 0.22 | 0.91 |
| Android fat (%) | 35.6 ± 0.69 | 35.1 ± 0. 70 | **0.02** | 0.90 | 0.33 | 0.94 |
| Gynoid fat (%) | 35.5 ± 0.76 | 35.3 ± 0.77 | 0.06 | 0.83 | 0.25 | 0.08 |
| Android fat % to gynoid fat % ratio | 1.03 ± 0.02 | 1.02 ± 0.02 | 0.23 | 0.93 | 0.58 | 0.35 |
| Trunk fat (%) | 33.3 ± 0.64 | 33.2 ± 0.64 | 0.75 | 0.91 | 0.15 | 0.82 |
| Total lean mass (kg) | 37.7 ± 0.74 | 38.0 ± 0.75 | **<0.0001** | 0.79 | 0.20 | 0.40 |
| Whole body total BMD (g/cm^2^) | 1.05 ± 0.01 | 1.05 ± 0.01 | 0.19 | 0.21 | 0.35 | 0.97 |

^1^ Refers to the treatment by time interaction in the model without gender as a fixed factor. ^2^Model with the gender, time and treatment included and testing the gender x time x treatment interaction. Bold values indicate p<0.05.

**Supplementary Table 3.** Frequency (n (%)) of the participants in the different categories of bone health status defined using T scores. T scores* less than or equal to -2.5 are defined to be osteoporosis, T scores between -2.5 and -1 are defined to be osteopenia and T scores greater than or equal to -1 are defined to be normal. The descriptive table below was based on those with complete data both at week 0 and week 8 (n=128).

| week 0 | | | | week 8 | | | |  |
| --- | --- | --- | --- | --- | --- | --- | --- | --- |
| Treatment group | Osteoporosis (%) | Osteopenia (%) | Normal (%) | Treatment group | Osteoporosis (%) | Osteopenia (%) | Normal (%) | *P^#^* |
| ROO | 5 (11.1) | 19 (42.2) | 21 (46.7) | ROO | 6 (13.3) | 19 (42.2) | 20 (44.4) | 0.50 |
| BO1 | 8 (18.6) | 11 (25.6) | 24 (55.8) | BO1 | 8 (18.6) | 12 (27.9) | 23 (53.5) | >0.99 |
| BO2 | 7 (17.5) | 19 (47.5) | 14 (35.0) | BO2 | 5 (12.5) | 23 (57.5) | 12 (30.0) | >0.99 |
| Pooled^ | 20 (15.6) | 49 (38.3) | 59 (46.1) | Pooled | 19 (14.8) | 54 (42.2) | 55 (43.0) | - |

^Pooled over the treatment groups within the respective weeks. ^#^ Unadjusted P-value from sign test conducted separately within each treatment group, α is 0.0167.

* As per Ministry of Health (MoH) guidelines, Singapore: <https://www.moh.gov.sg/docs/librariesprovider4/guidelines/cpg_osteoporosis.pdf>, accessed on 21^st^ Oct 2021.
